# Supplementary material for: Identification of distinct phenotypes related to benralizumab responsiveness in patients with severe eosinophilic asthma
Source: PLoS One. 2021 Mar 11;16(3):e0248305. doi: 10.1371/journal.pone.0248305 (PMC7951858; doi:10.1371/journal.pone.0248305)
Supplement: Attachment — Submitted filename: Response to reviewer ver.1.docx [file pone.0248305.s001.docx]

**Responses to the reviewers’ comments**

**Reviewer: #1**

**COMMENT 1**

The study population section is incomplete as basic characteristics are missing even from the Table 1. It is not clarified if patients have received other kind of biological in the past. What was the number of exacerbations in the previous 1 or 2 years? Besides allergic rhinitis, did they have nasal polyposis as well, and in what percentage?

**RESPONSE 1**

Thank you for the comment. In the revised manuscript, we have added the number of patients who had previous administration of other biologics at Table 1. We are afraid that information about previous exacerbation and nasal polyps is not available in this real-world cohort.

**COMMENT 2**

The cut off value of 173 IU/mL for IgE is rather arbitrary and high, as there are many references of 100 IU/mL as normal values. Furthermore, no information is provided about positive skin prick or Rast tests in order to better characterize the phenotype of severe allergic asthma.

**RESPONSE 2**

Thank you for the comment. According to the suggestion, in the revised manuscript, we have reanalyzed our data using the cut off value of 100 IU/mL, which showed no significant changes in the result. We agree that information about allergen specific IgE responses is important to better characterize each cluster of severe eosinophilic asthma; however, insufficient information about this in this real-world cohort did not allow us to do so.

**COMMENT 3**

There is no reference regarding changes in OCS use as almost 18 of the patients were under this treatment. OCS sparing is a major parameter of responsiveness to a biologic agent besides FEV1 improvement. The same applies for the number of exacerbations which should be included in the final analysis.

**RESPONSE 3**

Thank you for the comment. Although this study focused on the initial 4-month after starting benralizumab in a real-world setting, small but significant reduction in doses of maintenance OCS was noted during this period (Table 2). In terms of exacerbation, insufficient information about exacerbation history before starting benralizumab did not allow us to evaluate the effect of benralizumab.

**COMMENT 4**

Authors should make a comment comparing their results to those based on pool data of the initial phase III studies of Benralizumab (Bleecker ER, et al. Baseline patient factors impact on the clinical efficacy of benralizumab for severe asthma. Eur Respir J 2018; 52: 1800936) as there are both similarities and differences.

**RESPONSE 4**

Thank you for the comment. We have added a following sentence to the discussion in the revised manuscript.

“Pooled data analysis from the Phase III studies (SIROCCO and CALIMA) identified oral corticosteroid (OCS) use, nasal polyposis, pre-bronchodilator forced vital capacity (FVC), prior year exacerbations and age at diagnosis as baseline factors that influenced benralizumab efficacy (E1). In addition, the real-world effectiveness study of benralizumab also highlighted higher eosinophil counts, nasal polyposis, and adult-onset asthma as baseline characteristics associated with a superior response to benralizumab (E2). In the current study, in addition to elevated type-2 markers, lower FEV_1_ or OCS use appeared to be related to increased FEV_1_ response to benralizumab, which is in line with the previous studies. In contrast, age at disease onset was not related to the responsiveness in our study, which may be attributed to the rather older disease onset (57.8 years old) observed in our cohort.”

E1. Bleecker ER, Wechsler ME, FitzGerald JM, Menzies-Gow A, Wu Y, Hirsch I, et al. Baseline patient factors impact on the clinical efficacy of benralizumab for severe asthma. Eur Respir J 2018 Oct 18;52(4):1800936. doi: 10.1183/13993003.00936-2018.

E2. Joanne EK, Andrew PH, Jaideep D, Gráinne d'A, Abdel D, Cris R, et al. Real-World Effectiveness of Benralizumab in Severe Eosinophilic Asthma. Chest. Online ahead of print. DOI: <https://doi.org/10.1016/j.chest.2020.08.2083>

**COMMENT 5**

The conclusion should be more clear about which phenotypes relate to benralizumab responsiveness.

**RESPONSE 5**

Thank you for the comment. At the 1^st^ paragraph of the discussion, we have now described clearly that the greatest response was found in the distinct phenotype of severe eosinophilic asthma, which was characterized by modest increases in total IgE and FeNO relative to blood eosinophils with least exposure to smoking.

**MINOR　COMMENT**

1. Reference 1 does not support the first paragraph of the introduction section and the same applies for reference 7

2. References 24, 25 at the end of the first paragraph of introduction should be omitted

3. PSL abbreviation in Table 2 is not explained

4. Table 3 is rather confusing and most of the comparisons are not statistically significant, so it is suggested to be omitted. Table 5 could be omitted as Figure 3 is more informative

5. Statistical significant p values lower than 0,05 would better be written as p<0,001 instead of e.g. 3,1 x10-5

**RESPONSE to minor comment**

The above has been corrected as appropriate. In the revised manuscript, we omitted Table 5, but not Table 3. We believe that Table 3 has some importance of showing the baseline predictive factors for FEV_1_ improvement in response to 4-month benralizumab.

**Reviewer: #2**

**COMMENT 1**

Abstract: The paper is about identifying clusters of benralizumab treatment response. However, the Results summary does not clearly define which clusters were identified.

**RESPONSE 1**

Thank you for the comment. In the result section of abstract, we have now described clearly that the greatest response was found in the distinct phenotype of severe eosinophilic asthma, which was characterized by modest increases in total IgE and FeNO relative to blood eosinophils with least exposure to smoking.

**COMMENT 2**

Introduction is very generical and not informative. I would emphasize the importance of identifying clusters responding to benralizumab treatment outside clinical trials and to have information from real world settings.

**RESPONSE 2**

Thank you for the comment. We have revised the introduction accordingly.

**COMMENT 3**

Methods: Why not taking into account airway reversibility and post-bronchodilator FEV1? Please specify

**RESPONSE 3**

Thank you for the comment. We are afraid that we do not have sufficient information about airway reversibility and post-bronchodilator FEV_1_ in this cohort.

**COMMENT 4**

Results: Table 2 please describe all the abbreviations

**RESPONSE 4**

Thank you for the comment. We have revised Table 2 accordingly.

**COMMENT 5**

Discussion needs improvements. Please discuss in detail the published literature in light of the results of your research.

**RESPONSE 5**

We have added a following sentence to the 4th paragraph of the discussion in the revised manuscript.

“Pooled data analysis from the Phase III studies (SIROCCO and CALIMA) identified oral corticosteroid (OCS) use, nasal polyposis, pre-bronchodilator forced vital capacity (FVC), prior year exacerbations and age at diagnosis as baseline factors that influenced benralizumab efficacy (E1). In addition, the real-world effectiveness study of benralizumab also highlighted higher eosinophil counts, nasal polyposis, and adult-onset asthma as baseline characteristics associated with a superior response to benralizumab (E2). In the current study, in addition to elevated type-2 markers, lower FEV1 or OCS use appeared to be related to increased FEV1 response to benralizumab, which is in line with the previous studies. In contrast, age at disease onset was not related to the responsiveness in our study, which may be attributed to the rather older disease onset (57.8 years old) observed in our cohort.”

E1. Bleecker ER, Wechsler ME, FitzGerald JM, Menzies-Gow A, Wu Y, Hirsch I, et al. Baseline patient factors impact on the clinical efficacy of benralizumab for severe asthma. Eur Respir J 2018 Oct 18;52(4):1800936. doi: 10.1183/13993003.00936-2018.

E2. Joanne EK, Andrew PH, Jaideep D, Gráinne d'A, Abdel D, Cris R, et al. Real-World Effectiveness of Benralizumab in Severe Eosinophilic Asthma. Chest. Online ahead of print. DOI: https://doi.org/10.1016/j.chest.2020.08.2083

**COMMENT 6**

Discussion: tables and figures should not be referenced in the Discussion section

**RESPONSE 6**

Thank you for your comment. We have revised the manuscript accordingly.

**COMMENT 7**

Discussion: I would suggest adding a paragraph developing the potential usefulness of the study results and their generalizability.

**RESPONSE 7**

Thank you for your comment. Accordingly, we have added a sentence as the 3rd paragraph of the Discussion.
